# Supplementary figures and images for: LHAT-YOLO: Study on intelligent monitoring algorithm for helmets at construction sites
Source: PLoS One. 2026 Jan 28;21(1):e0339993. doi: 10.1371/journal.pone.0339993 (PMC12851489; doi:10.1371/journal.pone.0339993)

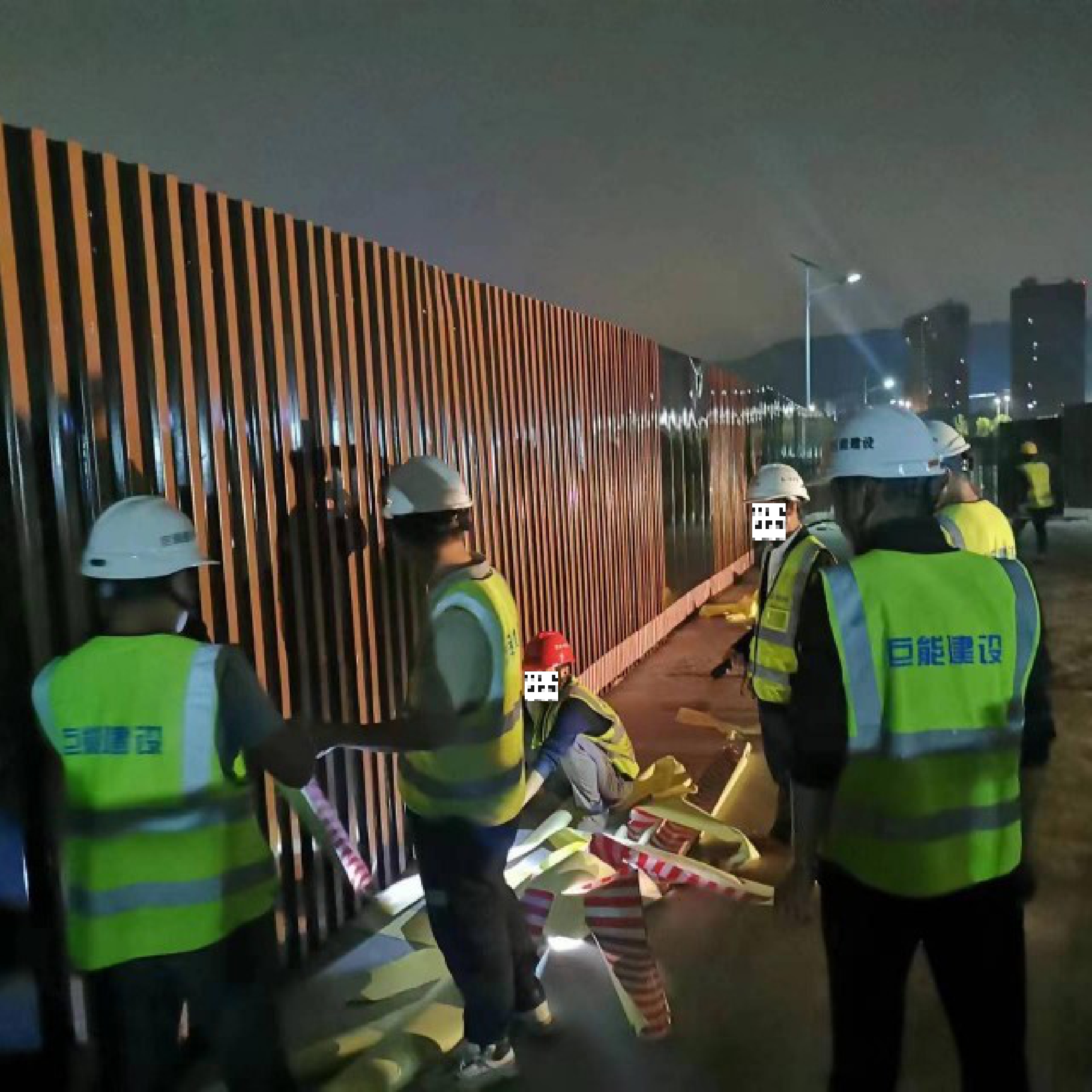

Supplement: S1 Data — (ZIP) [file pone.0339993.s001.zip › minimal data set/Figure7(a).jpg]

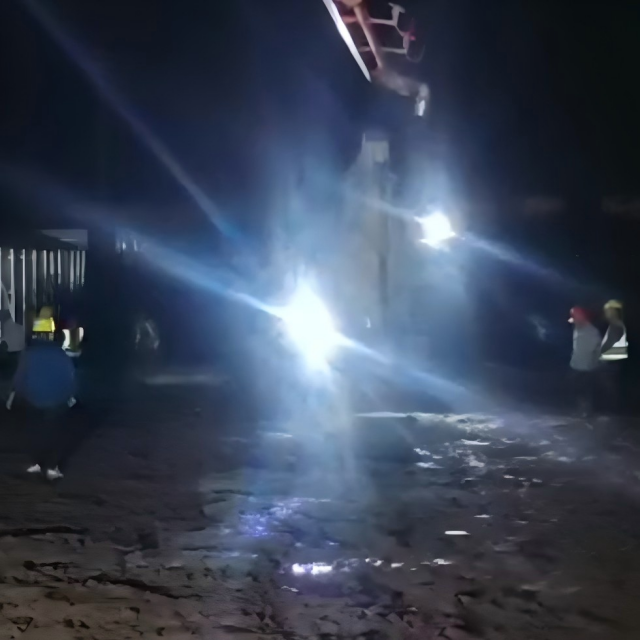

Supplement: S1 Data — (ZIP) [file pone.0339993.s001.zip › minimal data set/Figure7(d).jpg]

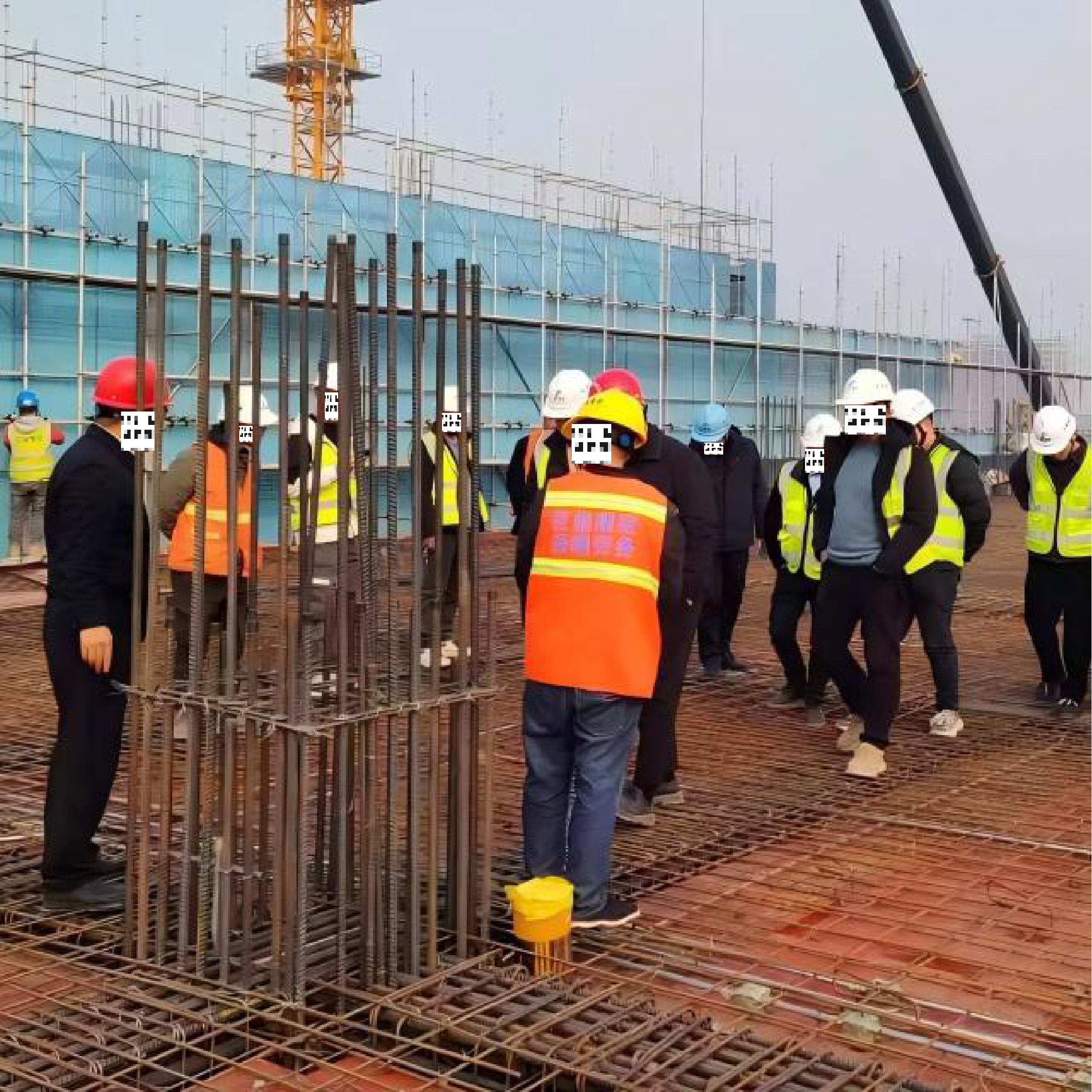

Supplement: S1 Data — (ZIP) [file pone.0339993.s001.zip › minimal data set/Figure7(g).jpg]

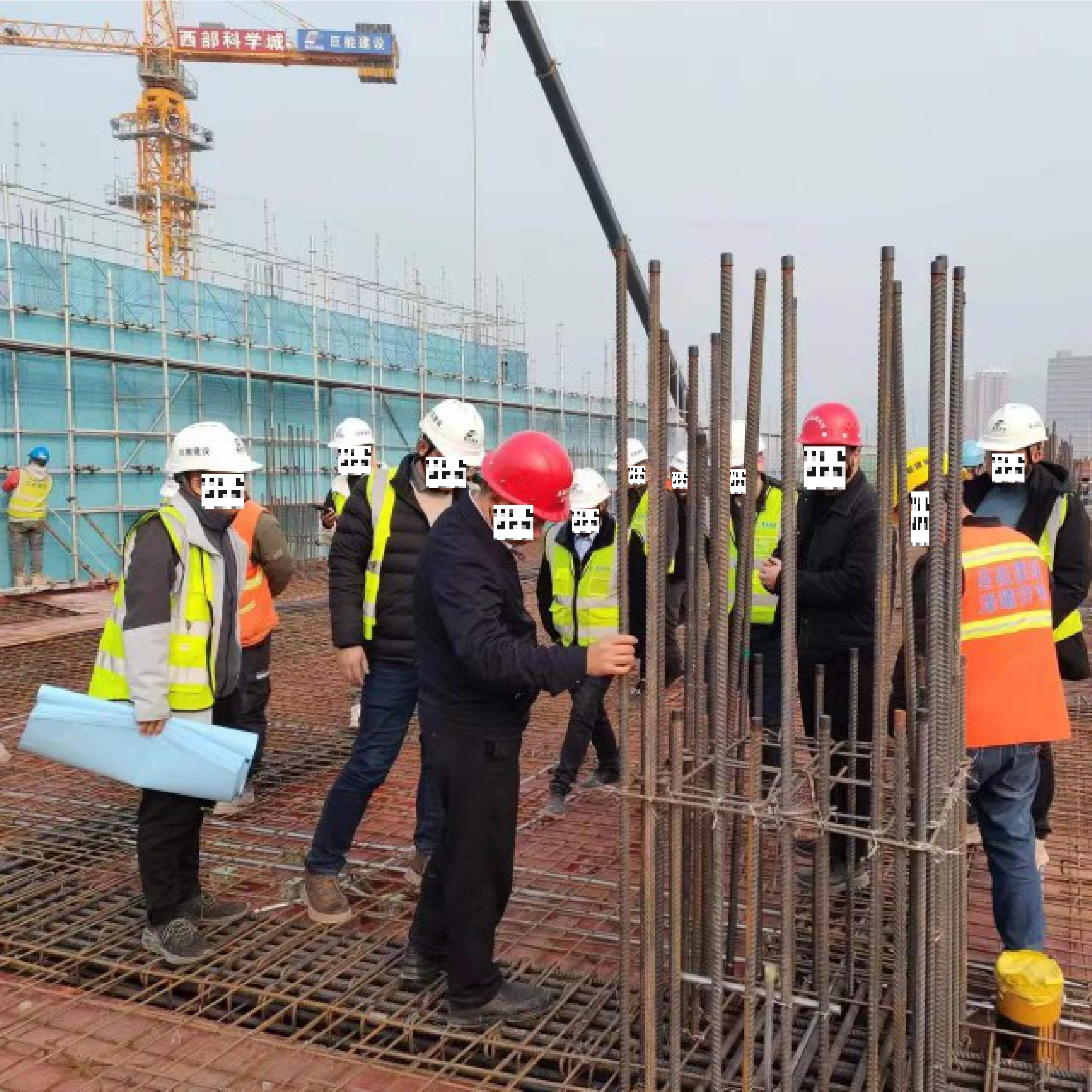

Supplement: S1 Data — (ZIP) [file pone.0339993.s001.zip › minimal data set/Figure7(j).jpg]

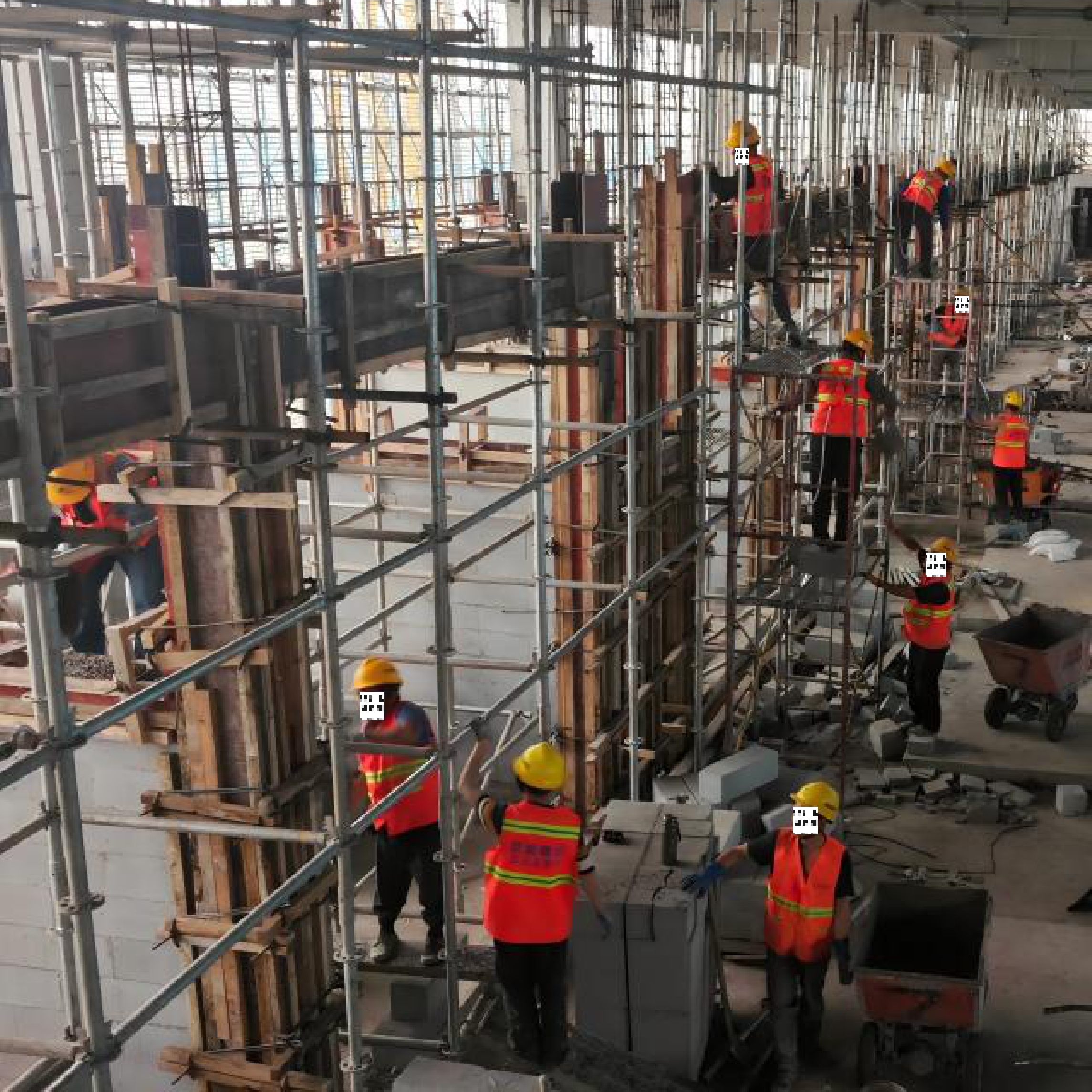

Supplement: S1 Data — (ZIP) [file pone.0339993.s001.zip › minimal data set/Figure9(a).jpg]

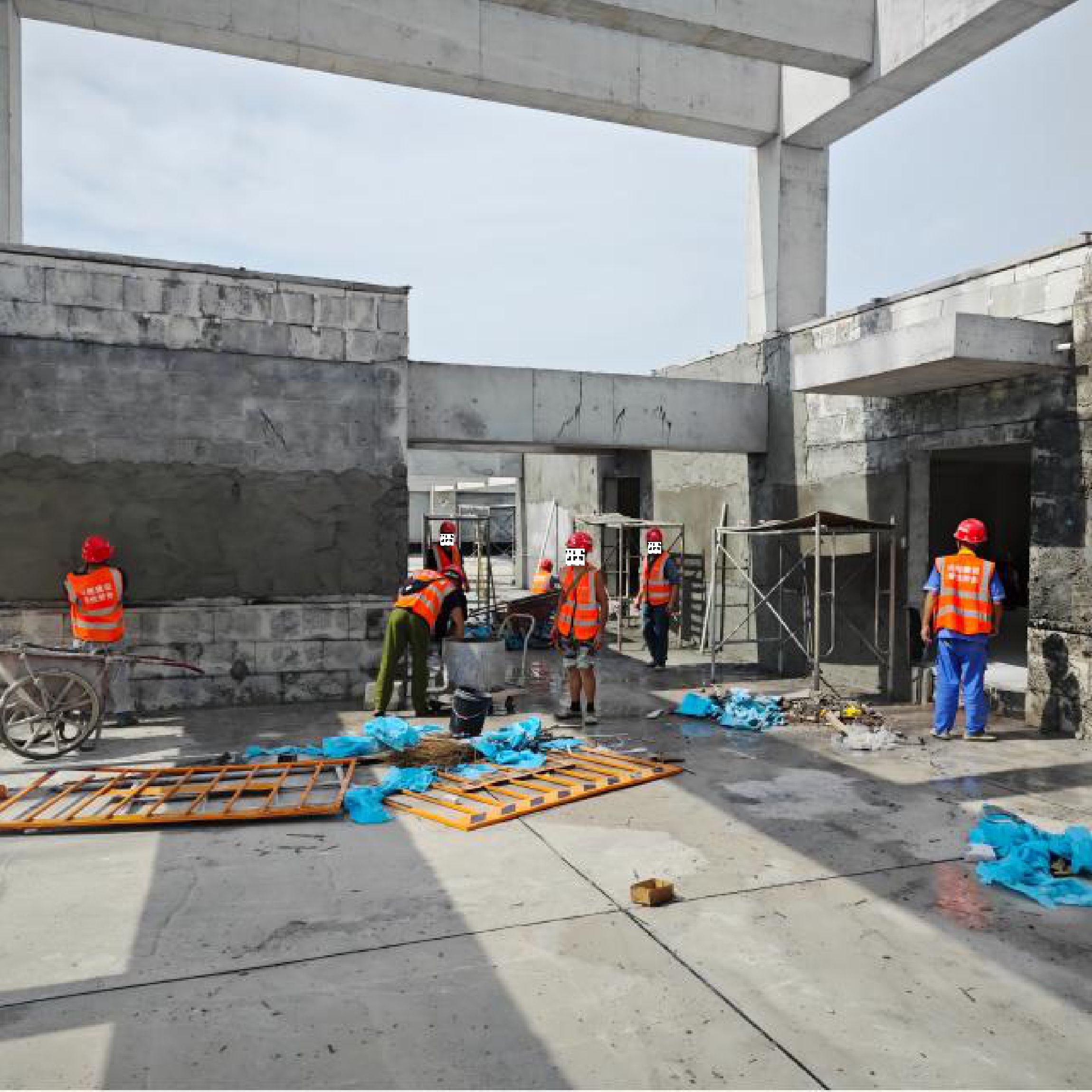

Supplement: S1 Data — (ZIP) [file pone.0339993.s001.zip › minimal data set/Figure9(d).jpg]

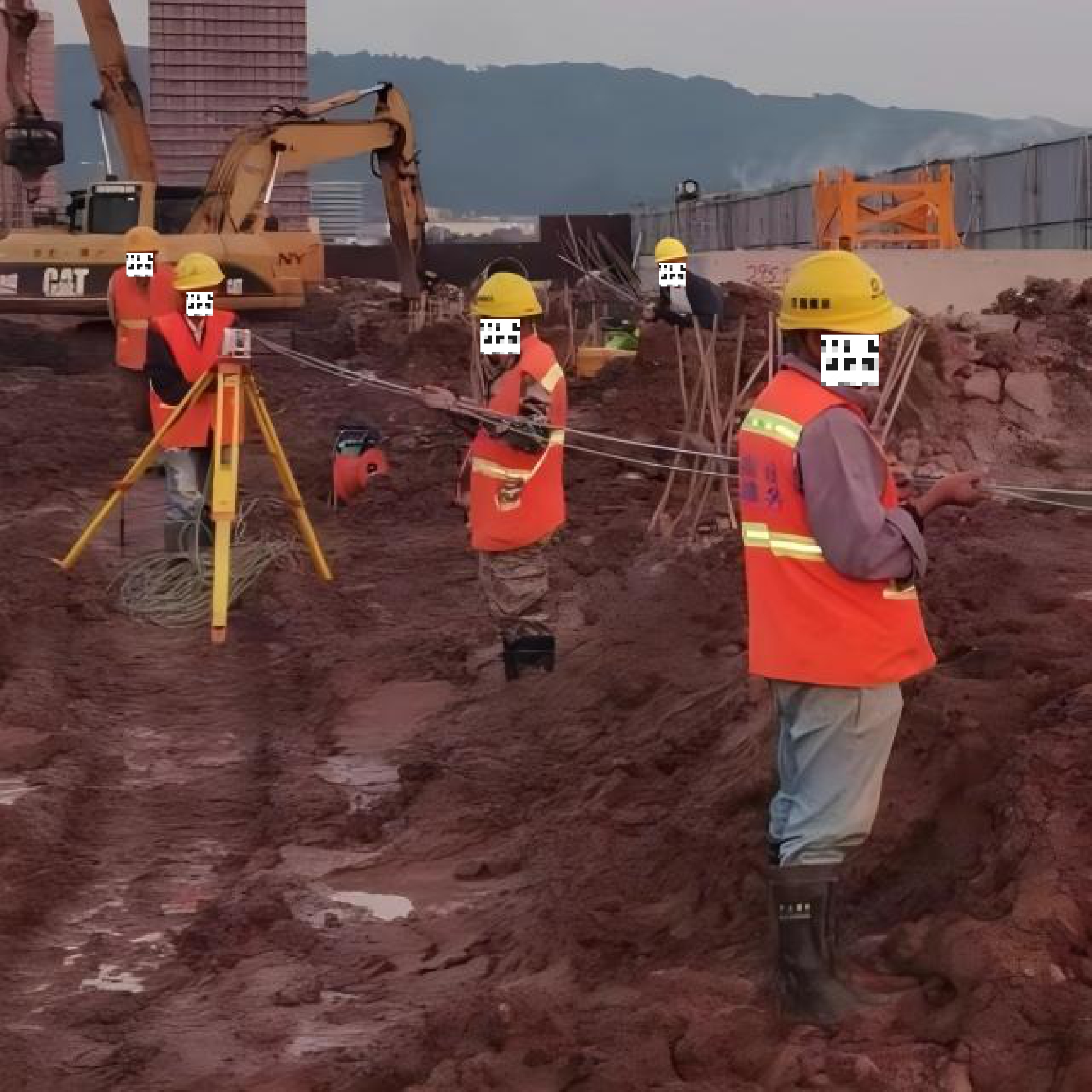

Supplement: S1 Data — (ZIP) [file pone.0339993.s001.zip › minimal data set/Figure9(g).jpg]

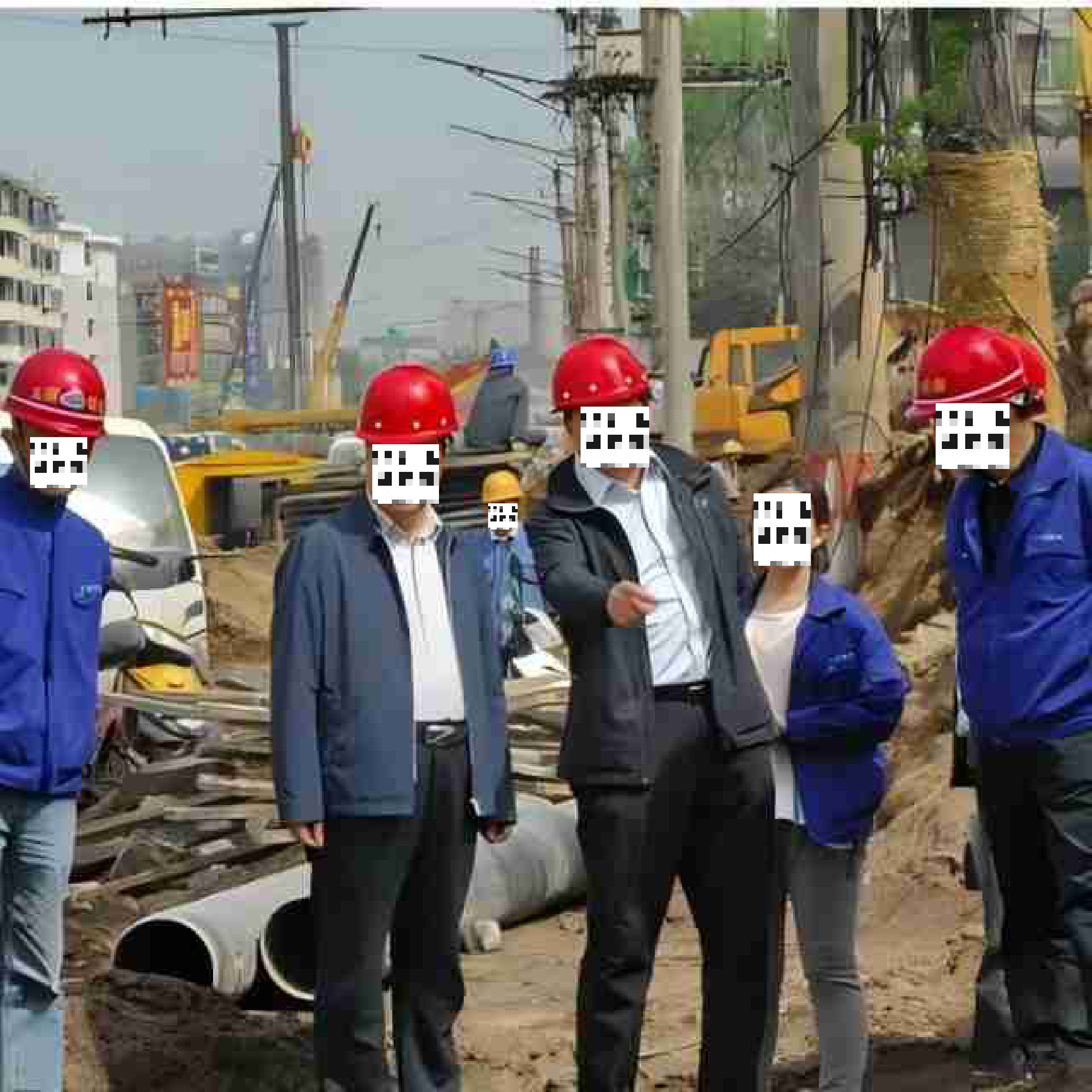

Supplement: S1 Data — (ZIP) [file pone.0339993.s001.zip › minimal data set/Figure9(j).jpg]
