# Supplementary material for: LHAT-YOLO: Study on intelligent monitoring algorithm for helmets at construction sites
Source: PLoS One. 2026 Jan 28;21(1):e0339993. doi: 10.1371/journal.pone.0339993 (PMC12851489; doi:10.1371/journal.pone.0339993)
Supplement: Attachment — Submitted filename: Response to Reviewers.docx [file pone.0339993.s003.docx]

**Manuscript Number: PONE-D-25-44125R1**

**Response to Reviewers**

Dear editors and reviewers,

Thank you for giving us the opportunity to submit a revised draft of the manuscript “**Application of LHAT-YOLO Model in Engineering: Research on Intelligent Monitoring Algorithm for Helmets at Construction Sites**” for publication in the ***Plos One***. The authors would like to thank the editors and reviewers for the time and effort they invested in providing valuable comments on our manuscript. We are deeply grateful to the reviewers for their insights and valuable suggestions for improvement. The authors have incorporated the reviewers' suggestions. These changes have been highlighted in the manuscript. Please see the point-by-point responses to the reviewers' comments in the blue text section below for specific changes.

**To Editors：**

It is a great honor to be recognized by the editors. The authors would like to thank the editors for their contributions in the process of revising the manuscript. Authors try to improve the quality of each part of the manuscript as much as possible. Once again, thank you for your review and support.

1. Please ensure that your manuscript meets PLOS ONE's style requirements, including those for file naming.

**Author response:** Thank you very much for your valuable comments. The authors have revised the manuscript according to the requirements of the editors and reviewers to ensure that the manuscript meets PLOS ONE's style requirements

2. In your Methods section, please provide additional information regarding the permits you obtained for the work. Please ensure you have included the full name of the authority that approved the field site access and, if no permits were required, a brief statement explaining why.

**Author response:** Thank you very much for your valuable comments. This manuscript has no dispute over its edition.

3. Please note that PLOS One has specific guidelines on code sharing for submissions in which author-generated code underpins the findings in the manuscript. In these cases, we expect all author-generated code to be made available without restrictions upon publication of the work. Please review our guidelines at https://journals.plos.org/plosone/s/materials-and-software-sharing#loc-sharing-code and ensure that your code is shared in a way that follows best practice and facilitates reproducibility and reuse.

**Author response:** Thank you very much for your valuable comments. All code and dataset materials can be obtained from the corresponding author.

4. We note that your Data Availability Statement is currently as follows: All relevant data are within the manuscript and its Supporting Information files.

Please confirm at this time whether or not your submission contains all raw data required to replicate the results of your study. Authors must share the “minimal data set” for their submission. PLOS defines the minimal data set to consist of the data required to replicate all study findings reported in the article, as well as related metadata and methods (https://journals.plos.org/plosone/s/data-availability #loc-minimal-data-set-definition).

For example, authors should submit the following data:

- The values behind the means, standard deviations and other measures reported;

- The values used to build graphs;

- The points extracted from images for analysis.

Authors do not need to submit their entire data set if only a portion of the data was used in the reported study.

If your submission does not contain these data, please either upload them as Supporting Information files or deposit them to a stable, public repository and provide us with the relevant URLs, DOIs, or accession numbers. For a list of recommended repositories, please see https://journals.plos.org/plosone/s/recommended-repositories.

If there are ethical or legal restrictions on sharing a de-identified data set, please explain them in detail (e.g., data contain potentially sensitive information, data are owned by a third-party organization, etc.) and who has imposed them (e.g., an ethics committee). Please also provide contact information for a data access committee, ethics committee, or other institutional body to which data requests may be sent. If data are owned by a third party, please indicate how others may request data access.

**Author response:** Thank you very much for your valuable comments. All code and dataset materials can be obtained from the corresponding author.

5. We note that Figure(s) 1, 6, 7, 8, 9 in your submission contain copyrighted images. All PLOS content is published under the Creative Commons Attribution License (CC BY 4.0), which means that the manuscript, images, and Supporting Information files will be freely available online, and any third party is permitted to access, download, copy, distribute, and use these materials in any way, even commercially, with proper attribution. For more information, see our copyright guidelines: http://journals.plos.org/plosone/s/licenses-and-copyright.

We require you to either (1) present written permission from the copyright holder to publish these figures specifically under the CC BY 4.0 license, or (2) remove the figures from your submission:

a. You may seek permission from the original copyright holder of Figure(s) 1, 6, 7, 8, 9 to publish the content specifically under the CC BY 4.0 license.

We recommend that you contact the original copyright holder with the Content Permission Form (http://journals.plos.org/plosone/s/file?id=7c09/content-permission-form.pdf) and the following text:

“I request permission for the open-access journal PLOS ONE to publish XXX under the Creative Commons Attribution License (CCAL) CC BY 4.0 (http://creativecommons.org/licenses/by/4.0/). Please be aware that this license allows unrestricted use and distribution, even commercially, by third parties. Please reply and provide explicit written permission to publish XXX under a CC BY license and complete the attached form.”

Please upload the completed Content Permission Form or other proof of granted permissions as an "Other" file with your submission.

In the figure caption of the copyrighted figure, please include the following text: “Reprinted from [ref] under a CC BY license, with permission from [name of publisher], original copyright [original copyright year].”

b. If you are unable to obtain permission from the original copyright holder to publish these figures under the CC BY 4.0 license or if the copyright holder’s requirements are incompatible with the CC BY 4.0 license, please either i) remove the figure or ii) supply a replacement figure that complies with the CC BY 4.0 license. Please check copyright information on all replacement figures and update the figure caption with source information. If applicable, please specify in the figure caption text when a figure is similar but not identical to the original image and is therefore for illustrative purposes only.

**Author response:** Thank you very much for your valuable comments. In fact, there are no copyright issues with the image data in the manuscript. The image data used was provided by the co-author of the manuscript, Haoran Jiang.

6. We note that Figure(s) 1, 6, 7, 8, 9 includes an image of a [patient / participant / in the study].

As per the PLOS ONE policy (http://journals.plos.org/plosone/s/submission- guidelines#loc-human-subjects-research) on papers that include identifying, or potentially identifying, information, the individual(s) or parent(s)/guardian(s) must be informed of the terms of the PLOS open-access (CC-BY) license and provide specific permission for publication of these details under the terms of this license. Please download the Consent Form for Publication in a PLOS Journal (http://journals.plos.org/plosone/s/file?id=8ce6/plos- consent-form-english.pdf). The signed consent form should not be submitted with the manuscript, but should be securely filed in the individual's case notes. Please amend the methods section and ethics statement of the manuscript to explicitly state that the patient/participant has provided consent for publication: “The individual in this manuscript has given written informed consent (as outlined in PLOS consent form) to publish these case details”.

If you are unable to obtain consent from the subject of the photograph, you will need to remove the figure and any other textual identifying information or case descriptions for this individual.

**Author response:** Thank you very much for your valuable comments. In fact, the image data in the manuscript does not have any copyright disputes. The image data used was provided by the co-author of the manuscript, Haoran Jiang. These images were all collected by the company at construction sites, and no permission documentation is required.

7. If the reviewer comments include a recommendation to cite specific previously published works, please review and evaluate these publications to determine whether they are relevant and should be cited. There is no requirement to cite these works unless the editor has indicated otherwise.

**Author response:** Thank you very much for your valuable comments. The authors will seriously consider whether all the literature is of reference value to ensure the rigor of scientific research.

**Reviewer #1**

The Manuscript " Application of LHAT-YOLO Model in Engineering: Research on Intelligent Monitoring Algorithm for Helmets at Construction Sites" conducts a study on the helmets detection algorithm based on Yolo11. Through comparisons with different Yolo algorithms and experiments on different datasets, the advantages of the improved algorithm are verified but the advantages are not clearly. Generally speaking, this paper has a certain degree of academic value, but the innovation is insufficient. The paper needs to be significantly revised in the following aspects.

**Author response:** We are very honored that our manuscript has received your recognition. The manuscript indeed still has many shortcomings. The authors will revise the manuscript according to your requirements. Below are our responses to the questions you raised. We hope that the authors' revisions and responses will meet with your approval. Thank you once again for taking the time out of your busy schedule to review our manuscript.

1. The title is too long and the first half of the title can be deleted. Generally, "study" should be used instead of "research" in the title.

**Author response:** Thank you very much for your valuable suggestions. The authors have removed the first part of the manuscript title as requested. Additionally, the term “study” has been changed to “research” in both the title and the main text of the manuscript. This will further enhance the readability of the manuscript. We appreciate your recommendations once again. The revised content is highlighted in red font in the manuscript and accompanied by annotations.

2. The last paragraph of the introduction does not sufficiently summarize the innovation. The overall innovation of the manuscript is insufficient, and the manuscript does not reflect the issue of systematic real-time performance.

**Author response:** We sincerely appreciate your valuable suggestions. The authors have rephrased the final paragraph of the introduction to fully summarize the innovative contributions of the entire paper. Once again, thank you for your suggestions. The revised content is highlighted in red font in the manuscript and accompanied by annotations.

3. In Figure 5, the modification of YOLOV11 mainly lies in where it is located, but the representation in the figure is insufficient.

**Author response:** We sincerely appreciate your valuable comments. The authors have revised Figure 5 to highlight the improved aspects of the model. Additionally, they have provided a more detailed description of Figure 5 to emphasize these enhancements. Once again, thank you for your comments. This will further enhance the readability of the manuscript. The revised content is highlighted in red font in the manuscript and accompanied by annotations.

4. From the perspective of ablation experiments in Table 2, the improvement in algorithm performance is not obviously significant overall? And in the column of parameter quantity in Table 2, 6 should be an exponent.

**Author response:** Thank you very much for your valuable comments. The number 6 here indeed refers to an index, and the authors have already made the necessary revisions. Since the model underwent lightweight improvements primarily aimed at reducing training time and decreasing the number of parameters, the performance gains were not significant. Once again, we appreciate your suggestions. This will further enhance the readability of the manuscript. The revised content is highlighted in red font in the manuscript and accompanied by annotations.

5. In fact, there are many studies on safety helmet detection algorithms. This manuscript only compared with other Yolo algorithms in Table 3, and did not compare with other optimization algorithms based on Yolo. Therefore, it cannot fully reflect the innovation of the author's work.

**Author response:** Thank you very much for your valuable comments. The authors considered this issue prior to submitting the manuscript. However, since we did not utilize public datasets, we could not directly compare our results with those reported in other authors' papers. The dataset designed in the manuscript was specifically intended to train a model for detecting hard hats in complex construction environments. Applying this model to ordinary, simple datasets would likely lead to overfitting. Therefore, the authors did not compare their results with those from other papers. Nevertheless, your suggestion is highly constructive. We appreciate your suggestions once again.

6. The summary of the entire manuscript in the conclusion is not simple enough. The title is "Engineering Application ", but this point was not reflected at the end.

**Author response:** We sincerely appreciate your valuable comments. First, the authors have refined the content of the Conclusion section. Second, the authors have removed the first part of the manuscript title as requested to more accurately reflect the core focus of this study and emphasize the fundamental research nature of the manuscript. Once again, thank you for your suggestions. The revised content is highlighted in red font in the manuscript and accompanied by annotations.

**Reviewer #2**

This manuscript proposes LHAT YOLO, a lightweight variant of YOLOv11 for detecting hardhat use on construction sites. The authors replace parts of the YOLOv11 backbone with GSConv and introduce a new Fast Convolutional Detection (FCD) head that includes “difference convolutions” to emphasize local gradients. On a self compiled dataset of 24,726 images labeled “helmet” and “person,” they report 11% lower GFLOPs and 9.5% fewer parameters than YOLOv11n, with precision 93.95%, recall 88.99%, mAP@50 94.92%, and mAP@50–95 65.28%. Qualitative examples suggest better robustness at night, under occlusion, and in clutter. However, this manuscript must be improved more for publication.

**Author response:** First, we sincerely thank you for your contribution to reviewing this manuscript. The authors are deeply honored that you have taken the time to evaluate our work. We will make further improvements to the manuscript as per your suggestions. Once again, thank you for your patience and thorough review.

1. Abstract is missing thorough review of applications of the most recent AI techniques and drones in safety helmet detection, for example, application of Transformer in M.Z. Shanti et al. Enhancing worker safety at heights: A deep learning model for detecting helmets and harness using DETR architecture, IEEE Access, 2025, pp151788-151802, and application of drones in M.Z. Shanti et al. Real-time monitoring of work-at-height safety hazards in construction sites using drones and deep learning, Journal of Safety Research, 2022, 83, pp364-370.

**Author response:** Thank you very much for your valuable comments. The manuscript indeed lacks an in-depth review of the latest artificial intelligence technologies and the application of drones in the field of safety helmet inspection. The authors have revised the abstract and introduction as requested and added new references. This will further enhance the rigor of the manuscript. Once again, thank you for your comments. The revised content is highlighted in red font in the manuscript and accompanied by annotations.

2. The FCD description is conceptual, but reproducibility requires exact architectural details: in/out channels and strides per branch, kernel sizes, the composition and ordering of CDC/HDC/VDC/ADC, normalization types, activation function placement, and how outputs are fused (Fig. 4a–b; pages 13–14). Please provide a layer by layer specification or an open source config file. Also, cite the original “Central Difference Convolution” work and any sources for the horizontal/vertical/angular variants referenced on lines 134–136.

**Author response:** Thank you very much for your valuable comments. The authors have revised this section according to your suggestions, and the complete FCD code can be provided by the corresponding author upon request. Additionally, the authors have cited the original paper on “Central Difference Convolution” as requested. This will further enhance the rigor of the manuscript. We extend our gratitude to you once again. The revised content is highlighted in red font in the manuscript and accompanied by annotations.

3. The manuscript repeatedly refers to adding a “complex object detection layer” to YOLOv11 (lines 140–146). Please specify whether you added an extra detection scale (e.g., P2 with stride 4 or 8) and how it interfaces with the neck. Include final detection strides, feature map sizes for 640×640 input, and the number of heads. Sentence to amend: “Due to the complex and changing environment at the construction site. The study chose to add a complex object detection layer…” Proposed: “To better detect small and occluded objects, we add an additional detection scale at [specify level, e.g., P2, stride 8], fed by [neck block(s)], yielding [K] detection heads at strides [s1, s2, s3,(s4)].”

**Author response:** Thank you very much for your valuable comments. To enhance the model’s capability of detecting fine-grained and small-scale objects, we introduce an additional detection scale, P2 (stride = 4), into the YOLOv11 detection head. As shown in Fig. X, the P2 feature map is generated by upsampling the P3 feature by a factor of 2 and concatenating it with the shallow backbone feature (stage-2 output, stride = 4). The fused feature passes through a C3k2 module to refine the local context and is then forwarded to the shared detection head (FCD). The modified head therefore produces predictions from four scales (P2–P5), covering a wider range of object sizes compared with the original three-scale YOLOv11 (P3–P5). Besides, The authors have revised the sentence “Due to the complex and changing environment at the construction site. The study chose to add a complex object detection layer...” as per your request. This will further enhance the readability of the manuscript. We extend our gratitude to you once again. The revised content is highlighted in red font in the manuscript and accompanied by annotations.

4. On lines 72–75, GFLOPs is described as “the number of floating point operations performed per second by the model.” That defines FLOPS (rate), not FLOPs (count). Please correct to “theoretical number of floating point operations per forward pass (at 640×640).” Sentence to amend: “GFLOPs refers to the number of floating point operations performed per second by the model.” Proposed: “GFLOPs denotes the number of floating point operations per forward pass at the specified input size; we report theoretical complexity estimated from the architecture.”

**Author response:** Thank you very much for your valuable comments. The authors have revised the statement “GFLOPs refers to the number of floating point operations performed per second by the model.” to “GFLOPs denotes the number of floating point operations per forward pass at the specified input size; we report theoretical complexity estimated from the architecture.” as requested. This change will further enhance the readability of the manuscript. We extend our gratitude to you once again. The revised content is highlighted in red font in the manuscript and accompanied by annotations.

5. The paper lists OS/GPU/CPU and two hyperparameters but omits batch size, optimizer (SGD/AdamW), scheduler (cosine, one cycle), momentum/β, label smoothing, augmentations (Mosaic/MixUp/HSV/flip), EMA, NMS IoU and confidence thresholds, loss composition, number of epochs, and random seeds (lines 91–95). Please add these details and provide a public repository with training scripts and model weights.

**Author response:** We sincerely appreciate your valuable comments. The authors have revised the manuscript according to your suggestions. However, considering the large number of parameters, we have supplemented the key parameters in Table 1, while the remaining parameters can be obtained from the code. The complete training script and model weights are available upon request from the corresponding author. These modifications enhance the rigor of the manuscript. Thank you once again for your contributions. The revised content is highlighted in red font in the manuscript and accompanied by annotations.

6. Provide the training performance of the model, e.g., the plots of loss vs. epochs.

**Author response:** Thank you very much for your valuable comments. The authors have revised the manuscript according to your suggestions. The training performance of the model is now detailed in the manuscript. These revisions significantly enhance the readability of the manuscript. We extend our gratitude to you once again. The revised content is highlighted in red font in the manuscript and accompanied by annotations.

7. Provide tests for various environmental and image conditions, i.e., foggy, blurred, etc.

**Author response:** Thank you very much for your valuable comments. Your suggestion is highly valuable, as testing under various environmental and image conditions can further evaluate the model's performance. However, simple blur enhancement cannot simulate real foggy conditions. Therefore, the authors plan to dedicate significant time to collecting an image dataset of foggy construction scenarios and will incorporate this consideration in future research. Additionally, the authors have acknowledged your suggestion in the Conclusion section as one of the directions for future work. Once again, we extend our sincere gratitude to you. The revised content is highlighted in red font in the manuscript and accompanied by annotations.

8. Figures and text mention modules such as C2PSA, PSABlock, C3k2, SPPF in Fig. 5 without definitions in the main text. Please include short descriptions and citations for each non standard component. Also, unify “heat map/hot map” to “heatmap” throughout, e.g., lines 184–186 and lines 203–204. Sentence to amend: “Similarly, the hot map results also show…” Proposed: “Similarly, the heatmap results show…”.

**Author response:** Thank you very much for your valuable comments. The authors have made further revisions to the manuscript content as per your request. The authors have added references to key modules. Most content is detailed in the YOLOv11 paper, which this paper also cites: 32. Khanam R, Hussain M. YOLOv11: An Overview of the Key Architectural Enhancements. Computer Vision and Pattern Recognition. 2024. <https://doi.org/10.48550/arXiv.2410.17725.> Besides, The terms “heat map/hot map” throughout the manuscript have been uniformly changed to “heatmap.” This modification significantly improves the manuscript's readability. We extend our gratitude to you once again.

9. There are several grammatical errors in methodological sentences. Example: “A ablation experiment was conducted…” (line 154). Sentence to amend: “A ablation experiment…” Proposed: “An ablation experiment…”. Also, “Due to the complex and changing environment at the construction site. The study chose to add…” (lines 140–142) should be one sentence with a clear subject. These occur in sections where precision matters for readers trying to reimplement the approach.

**Author response:** We sincerely appreciate your valuable comments. The authors have made further revisions to the manuscript content as per your requests. To minimize grammatical errors, the authors thoroughly reviewed the entire text, scrutinizing each sentence and word. These revisions significantly enhance the manuscript's readability. Thank you once again for your assistance.

10. Add the dataset size and the baseline(s) used for the 11%/9.5% reductions, and replace “However,” before the metric list with “Overall,” to avoid the implication of a trade off that the results do not show (Abstract lines 14–21).

**Author response:** Thank you very much for your valuable comments. The authors have made further revisions to the abstract content as per your request. They have added the dataset size to the abstract and changed “However” to “Overall.” These modifications significantly enhance the manuscript's readability. We extend our gratitude to you once again. The revised content is highlighted in red font in the manuscript and accompanied by annotations.

11. Provide examples where LHAT YOLO fails, not just YOLOv11 failures, to guide future work.

**Author response:** Thank you for your valuable comments. Thank you very much for your valuable comments. The authors have addressed the limitations of LHAT-YOLO in Figures 6 and 8. They also mention directions for further model improvements in the Conclusion section. This enhances the rigor of the manuscript. Once again, we sincerely appreciate your contributions. The revised content is highlighted in red font in the manuscript and accompanied by annotations.

12. Use “mAP@50” and “mAP@50–95” or “mAP50 / mAP50–95” consistently.

**Author response:** Thank you very much for your valuable suggestions. The authors have uniformly adopted “mAP50 / mAP50–95” throughout the manuscript as per your request. This change will further enhance the readability of the manuscript. We extend our gratitude to you once again. The revised content is highlighted in red font in the manuscript and accompanied by annotations.

We believe that these revisions have significantly improved the quality and readability of the manuscript. We would like to express our sincere gratitude for your time and effort in reviewing our work and providing invaluable guidance for its enhancement. We sincerely hope that these revisions meet the expectations of the esteemed reviewers and the editorial board. we look forward to the opportunity to contribute to the scholarly community through the ***PLOS ONE***.

Best wishes!

Corresponding author：Jun Wang

E-mail: junwang20252025@163.com
